# Arsenic and Infectious Disease: A Potential Factor in Morbidity among Bangladeshi Children

**DOI:** 10.1289/ehp.119-a218a

**Published:** 2011-05

**Authors:** Angela Spivey

**Affiliations:** **Angela Spivey** writes from North Carolina about science, medicine, and higher education. She has written for *EHP* since 2001 and is a member of the National Association of Science Writers

Lower respiratory tract infection (LRTI) and diarrhea are two of the most common causes of morbidity and mortality in children under 5 years old, especially in low-income countries. A new prospective cohort study of the link between these types of infections and arsenic exposure revealed a dose-dependent increase in LRTI and diarrhea in relation to maternal arsenic exposure **[*EHP*** **119(5):719–724; Rahman et al.]**.

Earlier studies linked prenatal arsenic exposure to increased risk of infant mortality, and infectious disease has been suggested as a potential underlying cause in these deaths. No epidemiologic studies have been conducted to support that explanation, but there is evidence from a few animal and human studies that arsenic may cause immunosuppression.

The current study included 1,552 live-born infants of women enrolled during 2002–2004 in Matlab, Bangladesh. Arsenic exposure was assessed by measuring inorganic arsenic in maternal urine samples collected at gestational weeks 8 and 30. After birth, information on symptoms of LRTI and diarrhea in infants was collected at monthly home visits in which mothers recalled symptoms that had occurred over the previous 7 days.

The estimated relative risk of LRTI and severe LRTI increased by 69% and 54%, respectively, in the participants whose mothers had urinary arsenic concentrations in the highest quintile (262–977 μg/L), compared with offspring of mothers whose exposure was in the lowest quintile (less than 39 μg/L). The relative risk of diarrhea increased 20% for the highest-exposure group compared with the lowest-exposure group.

The authors observed that relative risks of LRTI and diarrhea increased irrespective of sociodemographic factors or nutritional status of the women. However, further evaluation is needed in a larger sample to evaluate possible effect modification, because the risks appeared more pronounced in low social strata.

Strengths of the study include the large sample size, the objective measure of arsenic exposure, and the followup of infants for one full year, which the authors say would reduce any influence of seasons on infection rates. Potential limitations include lack of measurements of infant exposure to arsenic, a lack of information about other potentially toxic substances in water and food, and reliance on mothers’ reports of disease symptoms and signs. Given the millions of people worldwide who drink well water with elevated arsenic concentrations, the study results could have serious public health implications and, taken together with previous studies showing health effects from this exposure, emphasize the need to reduce arsenic exposure via drinking water.

## Figures and Tables

**Figure:**
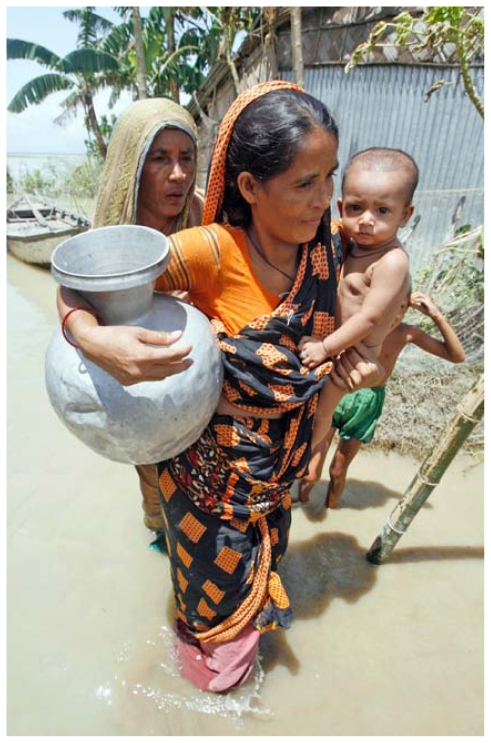
More than 50 million people in Bangladesh are believed to be chronically exposed to drinking water with arsenic concentrations exceeding the WHO standard of 10 μg/L.

